# Cryptic exon inclusion is a molecular signature of LATE-NC in aging brains

**DOI:** 10.1007/s00401-023-02671-0

**Published:** 2024-02-03

**Authors:** Mingee Chung, E. Kathleen Carter, Austin M. Veire, Eric B. Dammer, Jianjun Chang, Duc M. Duong, Nisha Raj, Gary J. Bassell, Jonathan D. Glass, Tania F. Gendron, Peter T. Nelson, Allan I. Levey, Nicholas T. Seyfried, Zachary T. McEachin

**Affiliations:** 1 Department of Cell Biology, Emory University, Atlanta, GA 30322 USA; 2 Laboratory for Translational Cell Biology, Emory University, Atlanta, GA 30322 USA; 3 Department of Biochemistry, Emory University, Atlanta, GA 30322 USA; 4 Department of Neuroscience, Mayo Clinic, Jacksonville, FL 32224 USA; 5 Department of Human Genetics, Emory University, Atlanta, GA 30322 USA; 6 Center for Neurodegenerative Diseases, Emory University, Atlanta, GA 30322 USA; 7 Department of Neurology, Emory University, Atlanta, GA 30322 USA; 8 Mayo Clinic Graduate School of Biomedical Sciences, Mayo Clinic, Jacksonville, FL 32224 USA; 9 Department of Pathology and Sanders-Brown Center On Aging, University of Kentucky, Lexington, KY 40536 USA

**Keywords:** Alzheimer’s disease, LATE-NC, Cryptic exons, TDP-43

## Abstract

The aggregation, mislocalization, and phosphorylation of TDP-43 are pathologic hallmarks of several neurodegenerative diseases and provide a defining criterion for the neuropathologic diagnosis of Limbic-predominant Age-related TDP-43 Encephalopathy (LATE). LATE neuropathologic changes (LATE-NC) are often comorbid with other neurodegenerative pathologies including Alzheimer’s disease neuropathologic changes (ADNC). We examined whether TDP-43 regulated cryptic exons accumulate in the hippocampus of neuropathologically confirmed LATE-NC cases. We found that several cryptic RNAs are robustly expressed in LATE-NC cases with or without comorbid ADNC and correlate with pTDP-43 abundance; however, the accumulation of cryptic RNAs is more robust in LATE-NC with comorbid ADNC. Additionally, cryptic RNAs can robustly distinguish LATE-NC from healthy controls and AD cases. These findings expand our current understanding and provide novel potential biomarkers for LATE pathogenesis.

**Supplementary Information:**

The online version contains supplementary material available at 10.1007/s00401-023-02671-0.

## Introduction

Pathologic aggregates of TAR DNA-binding protein 43 kDa (TDP-43) is a common neuropathological finding observed in many neurodegenerative diseases [7, 9]. First observed in frontotemporal lobar degeneration (FTLD-TDP) and amyotrophic lateral sclerosis (ALS) [37], TDP-43 pathology is now known to co-exist with other types of brain pathologies [7].

The most common subtype of TDP-43 pathology, distributed specifically in limbic brain regions, tends to be observed in the aging brain [14, 29, 52]. This pathologic pattern is termed Limbic-predominant Age-related TDP-43 Encephalopathy neuropathologic changes (LATE-NC) [33]. In community-based autopsy cohorts, LATE-NC is present in almost 40% of cases over the age of 80 years [32]. LATE-NC is strongly associated with cognitive decline [22, 31, 33].

LATE-NC frequently co-occurs with other brain pathologies, including Alzheimer’s Disease (AD) [1, 2, 21, 51] and Lewy Body Dementia (LBD) [30]. In the most common scenario of comorbid pathologies, LATE-NC has been reported in 19–75% of pathologically and clinically confirmed AD neuropathologic change (ADNC) cases [8, 10, 17, 20]. As a rule, the presence of TDP-43 pathology is associated with more severe clinical symptoms. For example, TDP-43 positive ADNC cases had worse clinical and neuropathological findings, including greater cortical atrophy and more severe cognitive impairment [19, 20]. Although LATE-NC often co-exists with ADNC, approximately a quarter of cases lacking any ADNC have “pure” LATE-NC [32]. Importantly, LATE-NC and FTLD-TDP are two distinct entities distinguished by the extent of phosphorylated TDP-43 (pTDP-43) burden, observable clinical manifestations, and age at death [31, 34].

Despite recent progress in studies of TDP-43 pathobiology, many questions remain about molecular mechanisms. TDP-43 is a ubiquitously expressed nucleic acid binding protein with well-established roles in alternative splicing and mRNA stability. Normally localized to the nucleus, TDP-43 is mislocalized from the nucleus to the cytoplasm resulting in loss-of-nuclear TDP-43 function.

It was recently demonstrated that TDP-43 represses the inclusion of cryptic exons [24]. Cryptic exons are non-conserved, unannotated intronic sequences that are erroneously spliced into RNA transcripts [12, 38] Inclusion of these cryptic exons in transcripts can promote transcript degradation through activation of various RNA surveillance mechanisms or result in translation of novel protein isoforms [26]. In vitro studies utilizing gene silencing methods to reduce TDP-43 abundance in induced pluripotent stem cell-derived neurons (iPSNs) have identified several TDP-43 regulated cryptic exon splicing events in neuronally enriched transcripts, including *STMN2, UNC13A*, *KALRN*, and *ELAVL3* [6, 23, 25, 27]. These studies have implicated TDP-43 regulated cryptic exons in disease pathogenesis, specifically, truncated *STMN2* (t*STMN2*) and *UNC13A* [6, 23, 25, 27]. Both genes encode proteins important for neuronal health and function; Stathmin-2 (*STMN2*) is a member of the Stathmin family of microtubule-binding proteins that functions to regulate microtubule dynamics important for axonal outgrowth and repair [41, 44, 48], whereas Protein unc-13 homolog A (*UNC13A*) is a member of the UNC-13 protein family that plays a role in synaptic vesicle maturation and transmission [4, 5]. Though less studied in the context of TDP-43 proteinopathies, both Kalirin (*KALRN*) and ELAV-like protein 3 (*ELAVL3*) are important for neuronal homeostasis, playing functional roles in synaptic plasticity [40] and axonal maintenance [39], respectively.

The presence of TDP-43-regulated cryptic exon splicing events in human post-mortem brain has been investigated in ALS, FTLD-TDP, and more recently, AD [6, 13, 23, 25, 27, 43, 49]; however, studies investigating whether neuronally enriched cryptic exons accumulate in the most common TDP-43 proteinopathy, LATE-NC, are lacking. In this study, we demonstrated that TDP-43 regulated cryptic exons accumulate in the hippocampus of a large, neuropathologically well-characterized LATE-NC cohort. Although ADNC is a common co-occurring pathology with LATE-NC, our quantitative approach to assess LATE and AD neuropathologic changes allow us to uniquely assess the accumulation of cryptic exons in pure LATE-NC cases. This demonstrates that cryptic exons are a defining characteristic of TDP-43 pathology irrespective of other comorbid neuropathologic inclusions. Our findings have important implications for the role of aberrant RNA metabolism in TDP-43 proteinopathies but also, more broadly, the aging brain. These data will inform future therapeutic strategies and biomarker development efforts for LATE-NC.

## Materials and methods

### Neuropathological assessment

Human post-mortem tissue from the hippocampus of 91 cases was selected from the University of Kentucky AD Research Center autopsy cohort (Supplementary Table 1); details of the cohort, inclusion/exclusion criteria, etc., and clinical diagnostic workups were previously described [35, 45]. For the purposes of the present study, a convenience sample was selected of hippocampi snap-frozen using liquid nitrogen at the time of autopsy and stored in -80C freezers thereafter. Neuropathologic assessments were performed separately. The pTDP-43 antibody used was the 1D3 clone (Sigma-Aldrich, USA) [36]. Stage-based diagnoses of LATE-NC, ADNC, and other pathologies used consensus-based criteria [3, 11, 28, 34]. None of the included cases had FTLD-TDP or any other non-LATE-NC pathology.

### Lysate preparation

Tissue lysates were processed as previously described [18]. In brief, ~50mg tissue was homogenized in 8M Urea buffer (8M Urea, 30mM Tris pH 8.0, 1x HALT protease/phosphatase inhibitor). Lysates were sonicated at 25% amplitude for 3 cycles of 4s on/4s off. Bicinchoninic acid (BCA) assay was used to determine protein lysate concentration.

### Meso-scale discovery (MSD) immunoassay

Phosphorylated TDP-43 abundance was determined using a custom MSD immunoassay [42]. The capture antibody was a mouse monoclonal antibody that detects TDP-43 phosphorylated at serines 409/410 (1:500, no. CAC-TIP-PTD-M01, Cosmo Bio USA), and the detection antibody was a sulfo-tagged rabbit polyclonal C-terminal TDP-43 antibody (2 μg/mL, 12892-1-AP, Proteintech). Tissue lysates in 8M urea were diluted to the same concentration and volume using 8M urea buffer, and then further diluted to the same concentration and volume using Tris-buffered saline. Samples of equal volume were tested in duplicate wells of the assay plate, and the average of the duplicates is reported and used for statistical analyses. Response values corresponding to the intensity of emitted light upon electrochemical stimulation of the assay plate using the MSD QUICKPLEX SQ120 were acquired. These response values were background corrected by subtracting the average response values from corresponding control lysates.

### Immunohistochemistry

Paraffin embedded sections (8 µm) were deparaffinized in Histo-clear (National Diagnostics) and rehydrated in ethanol. Antigen retrieval was performed in ddH_2_O by steam heat for 30 min. Endogenous peroxidase activity was quenched using hydrogen peroxide and washed 3x in PBS-T. Tissue sections were blocked using serum-free protein block (Dako) for 1 h. Slides were incubated in primary antibodies for 45 min at R.T. and washed 3x in PBS-T. Primary antibodies used for IHC were rabbit anti-pTDP-43 (Cosmo Bio TIP-PTD-P02); mouse anti-pTau (PHF-1); mouse anti-β-amyloid (BioLegend 800,703). HRP-conjugated secondaries (Dako) were applied for 30 min at R.T. Peroxidase labeling was visualized with 3,3ʹ-diaminobenzidine (DAB). Sections were subsequently counterstained with Gill’s hematoxylin and blued in Scott’s tap water substitute.

### RNA extraction, quantitative PCR (qPCR), and reverse transcription PCR (RT-PCR)

RNA was extracted using the Quick RNA kit (Zymo Research) with a combined on-column DNase I digestion step. For RNA extraction from post-mortem tissue, ~30mg of tissue was homogenized in lysis buffer using a bullet blender tissue homogenizer (Next Advance). RNA lysates were cleared by spinning samples at 10,000g for 1 min. RNA extraction was performed as per the manufacture’s protocol. cDNA was obtained via RT-PCR using the High-Capacity cDNA Reverse Transcription Kit (Thermo). 1100 ng total RNA was used for cDNA reactions; cDNA was diluted 1:5 with nuclease free water. To quantify relative mRNA expression for the cryptic exons, qPCR was performed for each sample using custom TaqMan gene expression assays (Thermo) on a Quantstudio 6 Flex system (Applied Biosystems). For each sample, 2μL of cDNA, corresponding to 22ng of input RNA, were loaded per well in duplicate for qPCR. Custom TaqMan assays including Assay IDs and sequences are detailed in Supplementary Table 2. The ΔΔCt method was used to assess relative expression. Relative tissue RNA expression was normalized to *RPLP0*, *GAPDH*, and *CYC1*; specifically, the geometric mean of *RPLP0*, *GAPDH*, *and CYC1* Ct values was calculated and subtracted from the Ct value for each cryptic exon TaqMan tested to generate a sample specific ΔCt. The ΔCt values for the healthy control samples were averaged to generate an averaged control group ΔCt value (ΔCt_AVG_). ΔΔCt values were calculated by subtracting the ΔCt_AVG_ from the ΔCt of each sample. Relative expression was calculated using 2^(−ΔΔCt)^. Undetermined values were set to aA Ct value of 40. For reverse transcription PCR (RT-PCR) 3μL of cDNA was amplified using Platinum II HS MasterMix (Thermo) according to the manufacturer’s protocol. Primers were designed to span constitutive/cryptic exon junctions. STMN2 Fwd: CTGCACATCCCTACAATGGC; STMN2 Rev: CCTTGTCAACTGTGCCACAA; ELAVL3 Fwd: AAGCCATCAACACCCTCAA; ELAVL3 Rev: AGGGCACAGGTAGACACACC; UNC13A Fwd: TGCTGGTGAATGAATGAATGATT; UNC13A Rev: ACAATCTCCTGGGCTGTCTC; β-Actin Fwd: CAACCGCGAGAAGATGAC; β-Actin Rev: AGGAAGGCTGGAAGAGTG.

### BaseScope^™^ in situ hybridization (ISH) assay

BaseScope^™^ assay (ACDbio) was performed per the manufacturer’s protocol with minor modifications. In brief, paraffin-embedded sections (8 µm) were deparaffinized in Histo-clear (National Diagnostics), incubated in 100% ethanol, and dried out for 5 min at 60 °C. Endogenous peroxidase activity was quenched using hydrogen peroxide for 10 min at R.T. Slides were subsequently washed 2x in ddH_2_O. Antigen retrieval was performed in 1x RNAscope^®^ Target Retrieval buffer for 30 min in a steamer. Tissue sections were treated with Protease IV for 30 min at 40°C. Slides were rinsed 2 × in ddH_2_O and then incubated with a custom BaseScope^™^ probe for 2h at 40°C. The custom BaseScope^™^ probe was designed to specifically detect the truncated *STMN2* splice variant (Probe ID: Ba-Hs-STMN2-cryptic-1zz-st-C1). ISH signal amplification was performed using the BaseScope^™^ Detection Reagent Kit V2 kit. Slides were washed in 1x RNAscope^®^ wash buffer between each ISH AMP step. Slides were developed with BaseScope^™^ Fast Red substrate. Sections were subsequently counterstained with Gill’s hematoxylin and blued in Scott’s tap water substitute.

### Interpretability of a machine-learning classifier model based on the Shapley Additive exPlanations (SHAP) approach

To determine the best predictors for estimating a LATE diagnosis, a random forest classifier (sklearn v1.3.1 in python 3.11.4) was trained using a fivefold cross-validation to categorize individuals in one of three disease classes (AD, Control, and LATE) using the eight cryptic exons and two existing biomarkers. An exhaustive grid search was used to determine the best parameter set for the model. The number of trees was evaluated at 25 evenly spaced values ranging from 10 to 500 and maximum depth of trees was assessed every 5 trees from 30 to 60. The minimum number of samples required to split a node was assessed at 5, 10, 15, and 20 samples and the minimum number of samples required to be a leaf at 1, 2, 4, 8, and 16. All features were considered when looking for the best split which was assessed using the Gini impurity measure. For each set of parameters, the cross validator was repeated five times with different training and testing splits. The final parameter set was as follows: criterion = gini, max_depth = 40, min_sample_leaf = 2, min_samples_split = 15, n_estimators = 50, bootstrap = True. A separate test set was used to evaluate the model accuracy. The optimized model accuracy score was 88.9% (Supplementary Fig. 1).

Performance of the classifier model was assessed using the area under the true-positive and false-positive rate from the receiver-operating characteristic (ROC) curve. We compared the predictive performance of the cryptic exons to existing biomarker data (pTDP43, tau Ratio), the performance of the top three features as predicted by SHAP, and the combination of all features.

To aid in the interpretation of generated model, the SHapley Additive exPlanations (SHAP) methodology was used [47]. The SHAP approach helps discern the significance of each feature in the final model-predicted output. The SHAP feature importance was calculated using shap v0.42.1. SHAP results were visualized using the shap package built in plot generation and custom in-house code using seaborn v0.12.2 and matplotlib v3.8.0.

### Statistical analysis

Statistical analysis of qPCR data was performed using ordinary one-way analysis of variance (ANOVA). Pairwise comparisons across all groups were performed with Tukey’s post hoc analysis. Statistical analysis of qPCR data was performed on ΔΔCt values. Statistical analysis of MSD immunoassay data was performed using Kruskal–Wallis one-way analysis of variance. Pairwise comparisons across all groups were performed with Dunn’s post hoc analysis. Kendall’s *τ* rank correlation coefficient was used to assess the correlation of cryptic exons and MSD data.

## Results

### Donor demographics and characterization

Demographic data on included research participants, including age, sex, PMI, and co-pathologies, are summarized in Table 1 and shown for each case in Supplementary Table 1. ADNC was determined by assessing for the presence of Aβ and phosphorylated tau immunoreactivity according to the consensus criteria for the neuropathological diagnosis of ADNC as published by the National Institute of Aging and Alzheimer Association working group (NIA-AA criteria) [15]. Cases with pTDP-43 immunoreactivity in the hippocampus were designated LATE-NC. In this study, we grouped LATE cases with “No” or “Low” AD neuropathologic changes as well as a Braak Stage ≤ 2 to be LATE -ADNC. Of the 91 cases, based on neuropathological examination, there were 21 neurological control cases, 25 ADNC without LATE-NC (“AD”), 35 LATE-NC cases with ADNC (LATE-NC +ADNC), and 10 LATE-NC cases without ADNC (LATE-NC -ADNC) (Table 1; Supplementary Fig. 2).

**Table 1:** Demographic, neuropathological, and clinical characteristics of cases

	Control	AD	LATE-NC (-ADNC)	LATE-NC (+ ADNC)
Donors	21	25	10	35
% Female	61.9%	64.0%	50.0%	65.7%
Age at onset (yrs)^a^	N/A	73 (52–90)	80.5 (62–93)	86.5 (71–89)
Age at death (yrs)^a^	85 (68–94)	82 (62–94)	91.5 (79–101)	90 (73–100)
Survival (yrs)^a^	N/A	7.5 (2–24)	7 (4–11)	9 (3–21)
PMI (hrs)^a^	2.67 (1.50–4.83)	3.00 (1.33–4.92)	2.35 (1.75–3.35)	2.50 (1.33–6.0)
Brain weight (g)^a^	1216 (970–1495)	1150 (920–1140)	1137.5 (990–1429)	1100 (880–1360)
Braak stage (*n*)				
0–II	21	0	10	0
III–IV	0	0	0	5
V–VI	0	25	0	30
Thal Phase (*n*)				
0	4	0	2	0
1	4	0	4	0
2	3	0	2	2
3	6	1	0	7
4	1	3	1	7
5	2	18	0	19
CERAD (*n*)				
Not	18	0	6	0
Possible	3	0	4	3
Probable	0	3	0	8
Definite	0	22	0	24
ADNC severity (*n*)				
No	9	0	2	0
Low	12	0	8	1
Intermediate	0	1	0	9
High	0	24	0	25
% Hipp. sclerosis	0.0%	4.0%	90.0%	85.7%
MMSE^a^	29 (23–30)	22 (0–28)	23.5 (12–30)	17 (0–30)
APOE Status (*n*)				
e2/e3	5	0	5	5
e2/e4	0	1	0	3
e3/e3	13	5	5	11
e3/e4	3	13	0	12
e4/e4	0	4	0	3

*AD*  Alzheimer’s Disease, *ADNC*  AD Neuropathological Changes, *LATE* Limbic-predominant Age-related TDP-43 Encephalopathy, *Hipp. Sclerosis* Hippocampal Sclerosis, *MMSE* Mini-Mental State Examination

^a^Reported values represent median (range)

### Quantitative assessment of pTDP-43 and pTau pathology

**Fig. 1:**
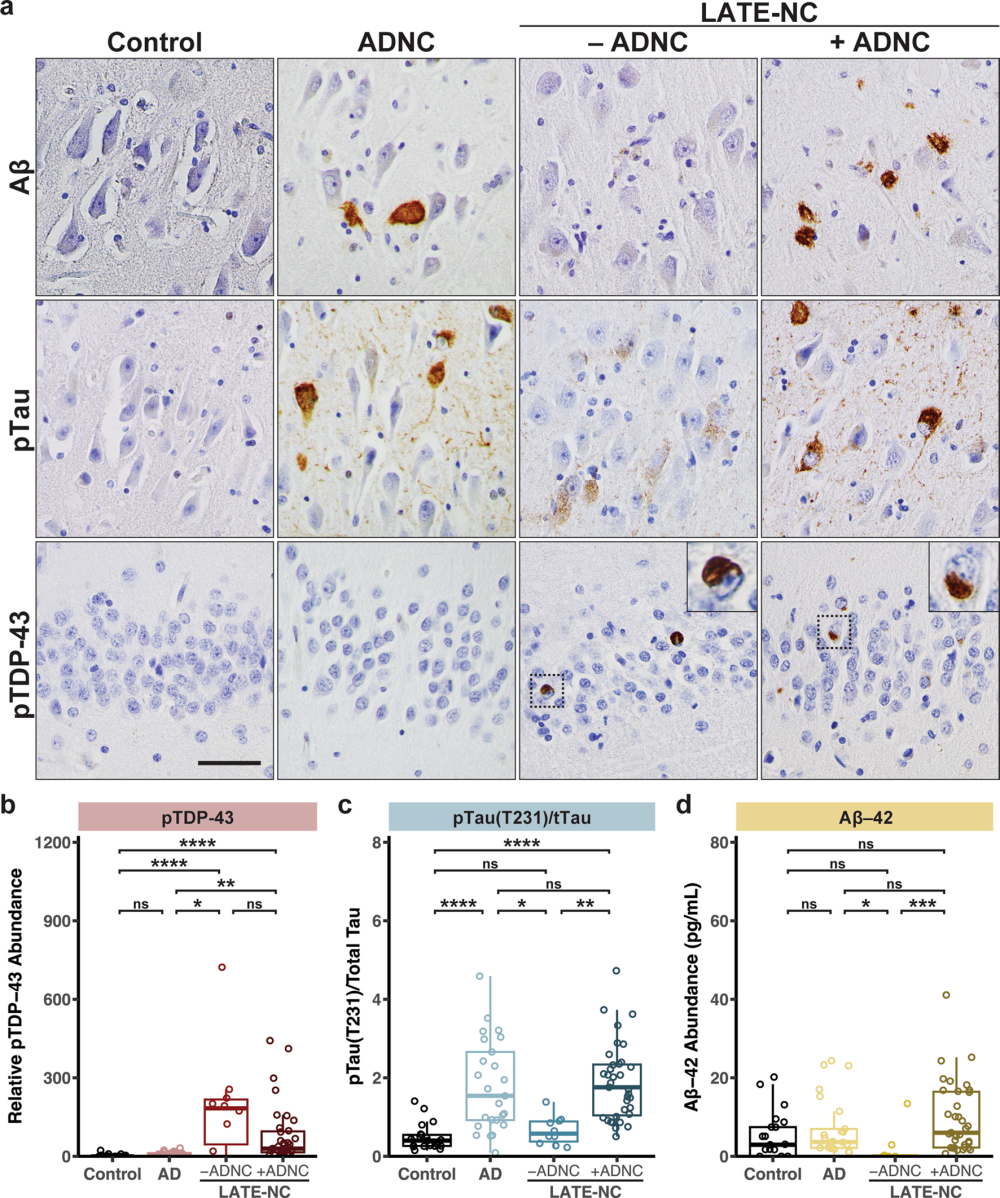
Immunohistochemical and quantitative immunoassay assessment of AD and LATE neuropathologic changes. a. Immunohistochemistry for pTDP-43, Aβ, and pTau in the hippocampus confirms ADNC and LATE-NC with or without ADNC. Scale bar = 50 μm**. b–d** Quantitative assessment of pTDP-43, phosphorylated tau (T231), and Aβ–42 in Control, AD, and LATE cases. pTau is normalized to total tau. *P < 0.05, **P < 0.01, ***P < 0.001, ****P < 0.0001, Kruskal-Wallis one-way analysis of variance followed by Dunn’s multiple comparison test

LATE-NC is defined at autopsy by the presence of pTDP-43 aggregates in distinct neuroanatomical regions; however, ADNC was often observed in LATE-NC cases (Fig. 1a). To further assess and quantify the extent of pTDP-43 pathology as well as AD associated neuropathological changes including phosphorylated Tau and Aβ-42 pathology, we performed meso-scale discovery (MSD) immunoassays on hippocampal tissue lysates (Fig. 1b-d; Supplementary Table 3). Consistent with the neuropathological examination by IHC, we observed robust and significant pTDP-43 abundance in the LATE-NC cohort with ADNC (+ADNC) and without ADNC (-ADNC) compared to the control and AD only cohorts (Fig. 1b). Notably, however, there was a broad range of pTDP-43 burden in both LATE-NC cohorts. To assess AD neuropathological changes in our cohorts, we quantified the abundance of Aβ-42, total tau and phosphorylated tau (T231), a tau post-translation modification observed in neurofibrillary tangles (Fig. 1c,d). For each sample, pTau (T231) abundance was normalized to the total tau abundance. Both AD and LATE-NC with ADNC cases had a significant increase of pTau (T231)/total Tau ratios (“pTau(T231)/tTau”), consistent with AD neuropathological changes observed by IHC (Fig. 1c). Additionally, we observed a strong correlation between pTau(T231)/tTau ratio with Braak Stage, but no correlation between pTDP-43 and Braak Stage (Supplementary Fig. 3a,b). Furthermore, we did not observe a correlation between pTau(T231)/tTau ratio and pTDP-43 abundance in the AD and LATE-NC cohorts (Supplementary Fig. 3c). Consistent with hippocampal sclerosis and neuronal loss in the hippocampus, we observed reduced abundance of total tau as measured by immunoassay in LATE-NC cases (Supplementary Fig. 3d).

### Assessment of cryptic exon accumulation in LATE-NC cases

**Fig. 2:**
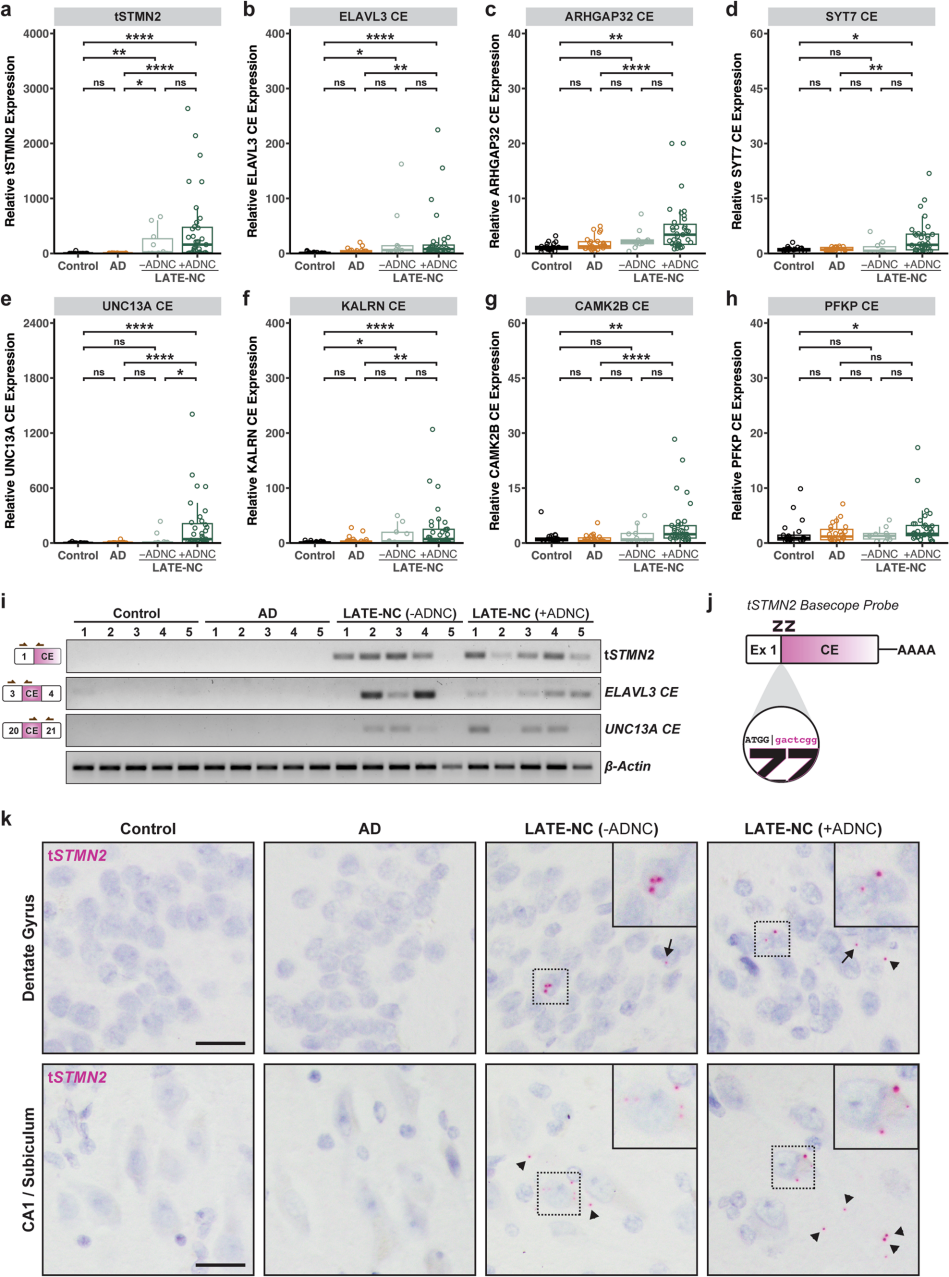
Inclusion of cryptic exons in LATE-NC. **a**–**h** Relative qPCR expression of several cryptic exons in the hippocampus of control (*n* = 21), AD (*n* = 25), and LATE cases with (+ ADNC; *n* = 35) or without (-ADNC; *n* = 10) AD neuropathologic changes shows robust expression of cryptic exons in LATE-NC cases compared to controls and AD. **i**. RT-PCR confirmation of cryptic exon inclusion in *STMN2*, *ELAVL3*, and *UNC13A* in LATE-NC but not Controls or AD. *Left*: Schematic of cryptic exon splice junctions; arrows represent relative position of PCR primers. **j** Schematic of a custom BaseScope^™^ in situ hybridization probe targeting the t*STMN2* Exon 1 – cryptic exon splice junction. **k** BaseScope^™^ in situ hybridization assay using a custom t*STMN2* probe shows robust t*STMN2* expression in the granule cell layer of the dentate gyrus and pyramidal neurons in the CA1 subfield and subiculum. Arrows point to intranuclear t*STMN2* RNA molecules; triangles point to extranuclear t*STMN2* RNA molecules. Sections were counterstained with hematoxylin. Scale bar = 25 μm. **a**–**h** *P < 0.05, **P < 0.01, ***P < 0.001, ****P < 0.0001, One-Way Analysis of Variance (ANOVA) followed by Dunn’s multiple comparison test

We next examined whether the inclusion of TDP-43 regulated cryptic exons was present in our AD and LATE-NC cohorts using quantitative PCR (qPCR). We designed several custom TaqMan primers targeting established cryptic exon splicing events (Supplementary Table 2). To ensure specificity of detection, our TaqMan probes were designed, such that hybridization of the forward and reverse primers spanned a cassette/cryptic exon junction and the reporter probe hybridized across the constitutive/cryptic exon junction. We observed significant inclusion of cryptic exons in several genes in the LATE-NC cohorts including t*STMN2*, *ELAVL3*, *UNC13A*, *KALRN*, *ARHGAP32*, *CAMK2B*, *PFKP*, and *SYT7* (Fig. 2a–h). The expression of several cryptic exons was significantly increased in both LATE-NC with ADNC and without ADNC cohorts including t*STMN2*, *ELAVL3*, and *KALRN* (Fig. 2a, b, f); however, cryptic splicing events in *UNCA13A*, *CAMK2B*, *SYT7*, *ARHGAP32*, and *PFKP* were more robustly detected in the LATE-NC +ADNC group compared to the -ADNC group (Fig. 2c–e, g, h). To confirm results obtained by qPCR, we performed reverse transcription PCR (RT-PCR) on several samples from each disease group using primers spanning constitutive/cryptic exon junctions for cryptic splicing events in *STMN2*, *ELAVL3*, and *UNC13A*; cryptic exons were detected only in LATE-NC cases but not Control or AD cases (Fig. 2i). As observed by qPCR, some cases exhibited variable inclusion of different cryptic splice events. To visualize the spatial and morphological distribution of the most robustly detected cryptic splicing event, t*STMN2*, we performed in situ hybridization (ISH) using a custom BaseScope^™^ probe that recognizes the STMN2 constitutive/cryptic exon splice junction (Fig. 2j). We observed t*STMN2* RNA molecules in the hippocampus of LATE-NC cases with or without ADNC (Fig. 2k). In the dentate gyrus, both nuclear and perinuclear t*STMN2* RNA molecules were detected in granule cells. In pyramidal neurons of the CA1 subfield and subiculum, t*STMN2* RNA molecules were detected in both the nucleus and cytoplasm; however, single neurons with several cytoplasmic t*STMN2* foci were often observed (Fig. 2k; Supplementary Fig. 4). Together, these findings demonstrate that TDP-43-regulated cryptic exon splicing events occur in the hippocampus of LATE-NC (Stage > 1) regardless of comorbid ADNC pathology.

### Cryptic exon accumulation correlates with pTDP-43 pathology in LATE-NC

**Fig. 3:**
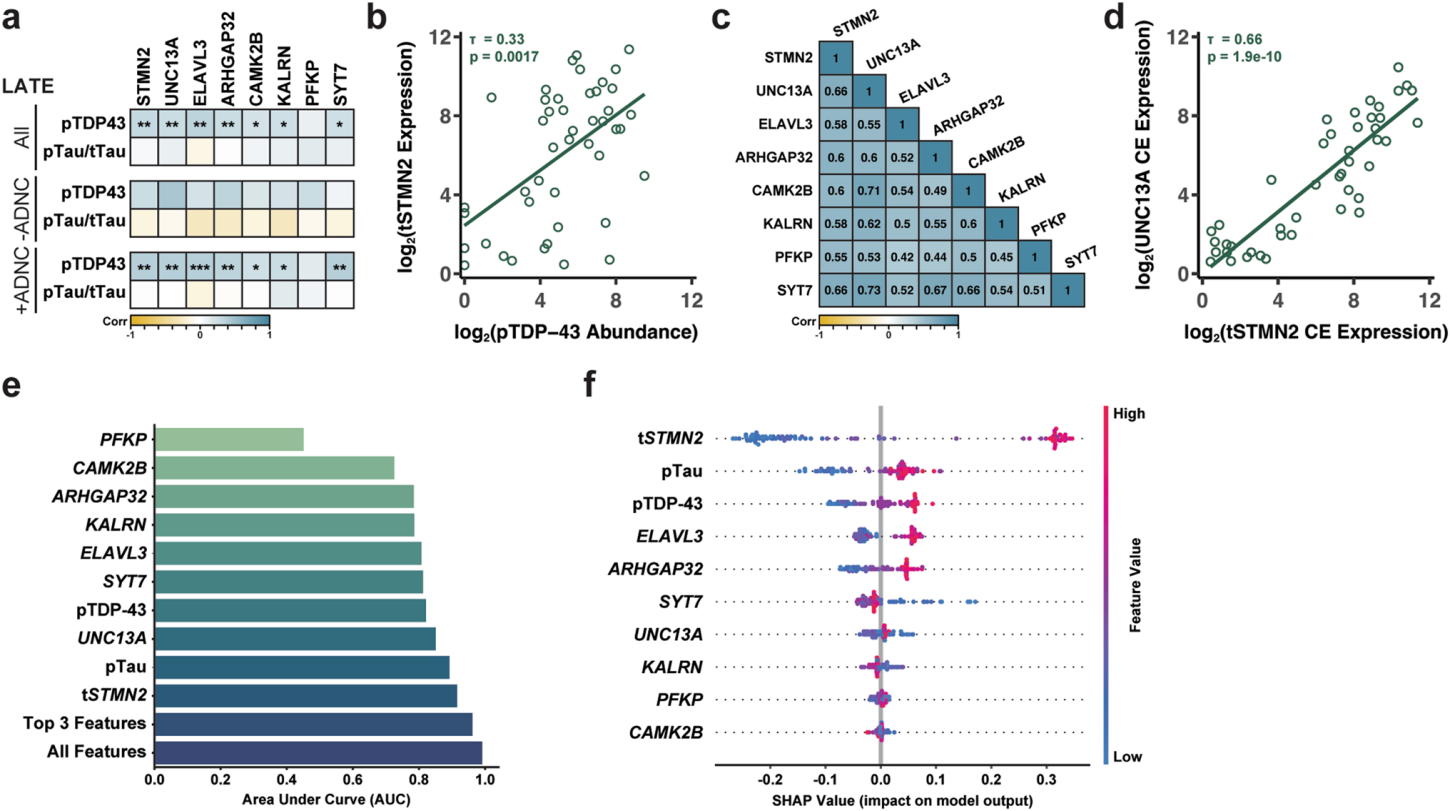
TDP-43 regulated cryptic exons correlate with pTDP-43 pathology in LATE-NC. **a** Cryptic exon expression correlates with pTDP-43, but not pTau, in LATE cases. **b** Scatterplots of tSTMN2 cryptic exon expression with pTDP-43 abundance. Cryptic exon expression and pTDP-43 abundance is log2 transformed. **c** Correlograms showing correlation coefficients between expression of several cryptic exons in LATE-NC. **d** Scatterplots of tSTMN2 and UNC13A cryptic exon expression. Cryptic exon expression is log2 transformed. **e** Barplot showing area under the curve (AUC) of the ROC curve for each cryptic RNA, pTau, and pTDP-43. Top 3 represents the AUC for the top three performing features; all represent the performance of our model if all features are included. **f** Beeswarm plot of SHAP values describing the impact of each feature on classifying LATE-NC cases. For each observation in the model, the calculated SHAP value (*x*-axis value) is represented by a single dot on each feature row. The color is indicative of the original value of the feature. A high value is indicated by red, whereas a low value by blue. Points are allowed to “swarm” together to indicate density in that region. Features were sorted by importance from top to bottom. **a**–**d** *P < 0.05, **P < 0.01, ***P < 0.001. Kendall’s *τ* Rank correlation

Previous studies have demonstrated that cryptic exon accumulation correlates with pTDP-43 pathology [43]. Following our finding that cryptic exons accumulate in the hippocampus of LATE-NC cases, we assessed whether these cryptic exon splicing events correlated with hippocampal pTDP-43 pathology. Indeed, we observed a significant correlation of pTDP-43, but not pTau(T231)/tTau, with all cryptic exon splicing events tested (Fig. 3a); however, when we assessed associations between pTDP-43 and cryptic exons based on LATE-NC with or without ADNC, we observed a more significant association between pTDP-43 pathology and cryptic exon accumulation in the LATE-NC +ADNC group. Of note, the t*STMN2* and *ELAVL3* cryptic exons had the strongest correlations with pTDP-43 pathology in the LATE-NC cohort (Fig. 3a,b). Additionally, we observed moderate, yet significant associations between all cryptic exon splicing events (Fig. 3c), suggesting that cases with increased occurrence of a particular cryptic exon splicing event (e.g., t*STMN2*) also have increased expression of other cryptic RNAs (e.g., *UNC13A*) (Fig. 3d).

### TDP-43-regulated cryptic exons distinguish LATE-NC from AD and controls

To further understand the robustness of the cryptic exon splicing events in distinguishing LATE-NC from AD and controls, a machine-learning classifier was developed. We optimized and trained a random forest classifier using the eight cryptic exon splicing events as well as pTDP-43 and pTau(T231)/tTau ratios obtained from bulk RNA and protein lysates to classify cases as either control, AD, or LATE-NC. LATE-NC group included both LATE-NC with or without ADNC. By training a classifier using the cryptic exons and the biomarkers, we can determine which measure is most predictive of LATE-NC (Supplementary Fig. 1a). t*STMN2,* pTDP-43, pTau(T231)/tTau*,* and *UNC13A* were the most important features of the model for distinguishing between the three diagnostic groups (Supplementary Fig. 1b).

Multiclass Receiver-Operator Characteristic (ROC) analysis was performed and the area under the curve (AUC) for each cryptic RNA, pTDP-43, and pTau(T231)/tTau was calculated (Fig. 3e). Given the imbalance in diagnostic group sizes (control *n* = 21, AD *n* = 25 LATE-NC *n* = 45), we reported the micro-averaged AUC which computes the metric independently for each class and then takes the average. Therefore, each class is weighed equally in the final value. Interestingly, the best-performing feature was t*STMN2* with an AUC of 0.91. This cryptic exon outperformed the more traditional marker of LATE-NC, pTDP-43, which distinguished the three classes with a reported AUC of 0.82. To assess the added benefit in including a cryptic exon in the determination of diagnostic group, the AUC of a model including the top three features that contributed to the overall predictive power was calculated. Including t*STMN2* with pTau(T231)/tTau, and pTDP-43 measures increased the AUC to 0.96 (Fig. 3e). When including all features, the AUC only marginally increases to 0.991. Thus, we show by including the t*STMN2* measurement with pTDP-43, we are able to more accurately predict the presence of LATE-NC in samples.

Shapley Additive exPlanation (SHAP) analysis was used to assess the contribution of each feature, i.e., the cryptic RNAs, pTDP-43, or pTau(T231)/tTau, to the predictive power of the model for each diagnostic group. Based on the concept of Shapley values from cooperative game theory, which assigns a value to each player in a game based on their contribution to the overall outcome, this statistical approach can be used to determine feature importance and aid in the interpretability of a given model [47]. In our study, this approach allows us to determine and rank the contribution of cryptic RNAs as well as established pathological protein hallmarks to classify LATE-NC. The SHAP plot for the feature contribution to the determination of LATE-NC is shown in Fig. 3f. Features were sorted by importance from top to bottom. Of note, t*STMN2* expression had the greatest impact to LATE-NC classification than any other feature including pTDP-43, suggesting that t*STMN2* expression is a robust and sensitive predictor of LATE-NC. The second most impactful cryptic RNA in determining LATE-NC diagnosis was *ELAVL3* (Fig. 3f). In contrast, high pTau(T231)/tTau and low pTDP-43, t*STMN2*, and *UNC13A* are the most impactful features to classify AD (Supplementary Fig. 1c).

## Discussion

In this study, we demonstrated that aberrant TDP-43-regulated cryptic exon splicing events occurred in the hippocampus of LATE-NC cases with or without comorbid ADNC. We found that t*STMN2* was the most robust and significant cryptic RNA expressed in LATE-NC. We also observed expression of several other cryptic RNAs, including *ELAVL3, UNC13A*, *KALRN*, *CAMK2B*, *ARHGAP32*, *SYT7*, and *PFKP*. The accumulation of cryptic exons in the hippocampus of Stage 2 LATE-NC occurred in parallel with pTDP-43 deposition in the hippocampus. An interesting finding in our study is that we observed accumulation of cryptic RNAs in LATE-NC cases without ADNC. These cases tended to be from older subjects at death (Supplementary Fig. 1). While these cases did not have observable ADNC or FTLD pathology, there was observable hippocampal sclerosis. It was previously demonstrated that hippocampal sclerosis is strongly associated with LATE-NC [1], which highlights an intriguing possibility that cryptic RNA accumulation could contribute to neuronal degeneration in hippocampal sclerosis seen in LATE-NC.

While LATE-NC with or without ADNC had similar pTDP-43 burden as measured by immunoassay, all cryptic RNAs measured had a greater accumulation in LATE-NC +ADNC, and several cryptic RNAs were significantly different only in the +ADNC group, including *UNC13A*, *CAMK2B*, *SYT7*, *ARHGAP32*, and *PFKP*. Despite not being significantly different as a group, a number of individual cases in the LATE-NC -ADNC cohort had increased cryptic RNA accumulation for several cryptic exons tested. It is possible that this difference in cryptic RNA accumulation between LATE-NC with and without ADNC is due to the limited sample size of our -ADNC cohort; however, it is also tempting to posit that neuronal dysfunction associated with ADNC potentiates RNA misprocessing events associated with TDP-43 pathology. This hypothesis is supported by the previous studies that have demonstrated that phosphorylated tau interacts with TDP-43 [50].

There was a moderate-to-strong correlation between cryptic RNA accumulation and pTDP-43 abundance in LATE-NC for the cryptic RNAs tested. Nevertheless, several cases with low pTDP-43 abundance, but high cryptic RNA accumulation were observed. Since inclusion of cryptic exons is hypothesized to result directly from the loss-of-nuclear TDP-43 function, it is possible that increased cryptic RNA accumulation in cases with low pTDP-43 is due to nuclear clearance of TDP-43 in a subset of neurons without overt pTDP-43 pathology. Notably, there were some ADNC only cases that had measurable cryptic RNA accumulation in several targets including *KALRN* and *ELAVL3*, although the expression was lower than LATE-NC; these cases could be similar to the LATE-NC pTDP-43 low/cryptic RNA high cases where there could be nuclear TDP-43 clearing without pTDP-43 pathology that results in cryptic exon inclusion.

TDP-43-regulated cryptic exon splicing events can serve as an RNA readout of a neuropathological event associated with neurodegeneration and thus have been proposed as a biomarker for various TDP-43 proteinopathies including ALS and FTLD [26]. Two recent studies demonstrated that some cryptic RNAs are translated to produce de novo peptides that can be detected by mass spectrometry or immunoassay in cerebrospinal fluid [16, 46]. Therefore, in this study, we evaluated whether cryptic RNAs can classify LATE-NC and thereby serve as a potential biomarker. Indeed, we found that several cryptic RNAs were able to discriminate LATE-NC from AD and controls. Interestingly, we found that t*STMN2* had the most significant impact on our random forest model to classify LATE-NC from AD and controls, suggesting that t*STMN2* cryptic RNAs may be a reliable molecular marker for an LATE-NC diagnosis.

Overall, our study provides evidence that cryptic exon splicing events are a neuropathological hallmark of LATE-NC with or without AD neuropathological changes and motivates future studies to understand the role of cryptic RNAs in LATE pathogenesis.

### Supplementary Information

Below is the link to the electronic supplementary material.

Supplementary file1 (PDF 3049 kb)

## Data Availability

All data are available from the corresponding author upon request.
