# Supplementary material for: Cryptic exon inclusion is a molecular signature of LATE-NC in aging brains
Source: Acta Neuropathol. 2024 Feb 3;147(1):29. doi: 10.1007/s00401-023-02671-0 (PMC10838224; doi:10.1007/s00401-023-02671-0)
Supplement: Supplementary file 1 — Supplementary file1 (PDF 3049 kb) [file 401_2023_2671_MOESM1_ESM.pdf]

Supplementary Materials for

Cryptic exon inclusion is a molecular signature of LATE-NC in aging brains

Chung *et al*.

Corresponding Authors: Zachary T. McEachin, zmceach@emory.edu
Nicholas T. Seyfried, nseyfri@emory.edu
Allan I. Levey, alevey@emory.edu

This File includes:

<u>Supplementary Figures:</u>

1. **Supplementary Figure 1** – Machine learning model confusion matrix and feature importance with resultant SHAP feature importance for two of the three diagnostic groups
2. **Supplementary Figure 2** – Boxplots of age and brain weight for all cases
3. **Supplementary Figure 3** – Associations of pTau(231) and pTDP-43
4. **Supplementary Figure 4** – Detection of tSTMN2 RNA molecules in LATE-NC by Basescope™ in situ hybridization assay

<u>Supplementary Tables:</u>

1. **Supplementary Table 1** – Detailed Neuropathological, Clinical, and Demographic Characteristics of Individual Cases
2. **Supplementary Table 2** – Custom Cryptic Exon TaqMan Probe Info
3. **Supplementary Table 3** – Information of Meso-Scale Discovery (MSD) Immunoassays

SUPPLEMENTARY FIGURES:

Supplementary Figure 1:

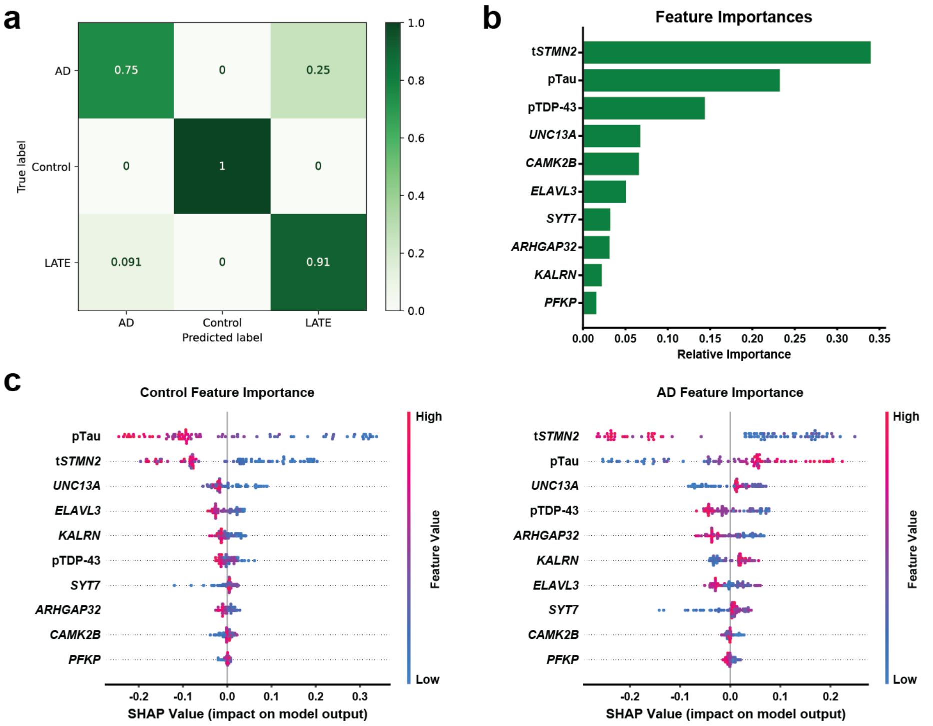

Supplementary Figure 1: Machine learning model confusion matrix and feature importance with resultant SHAP feature importance for two of the three diagnostic groups. a. Confusion matrix of random forest classifier to classify controls, AD, and LATE-NC. **b.** Barplot showing the feature importance of each feature in the Random Forest classifier. **c.** Beeswarm plot of SHAP values describing the impact of each feature on classifying control *(left)* and AD *(right)* cases.

Supplementary Figure 2:

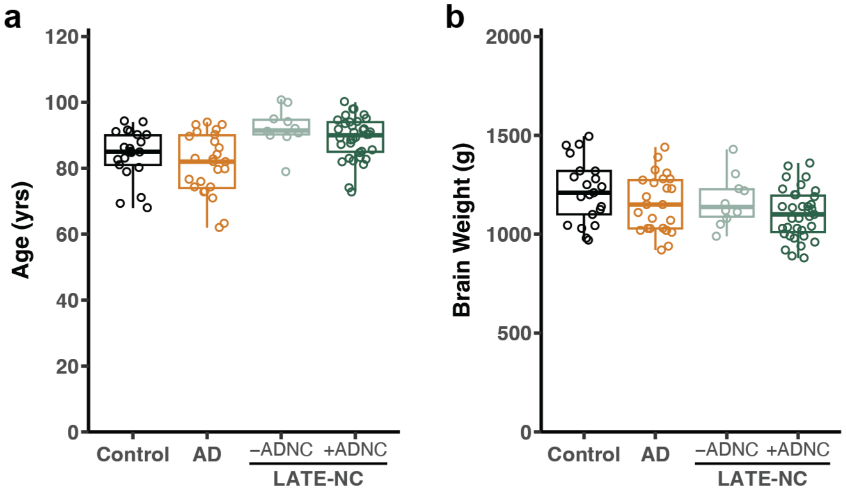

Supplementary Figure 2: Boxplots of age and brain weight for all cases. Boxplots of age **(a)** and brain weight **(b)** for control, AD, and LATE cases used in this study

Supplementary Figure 3:

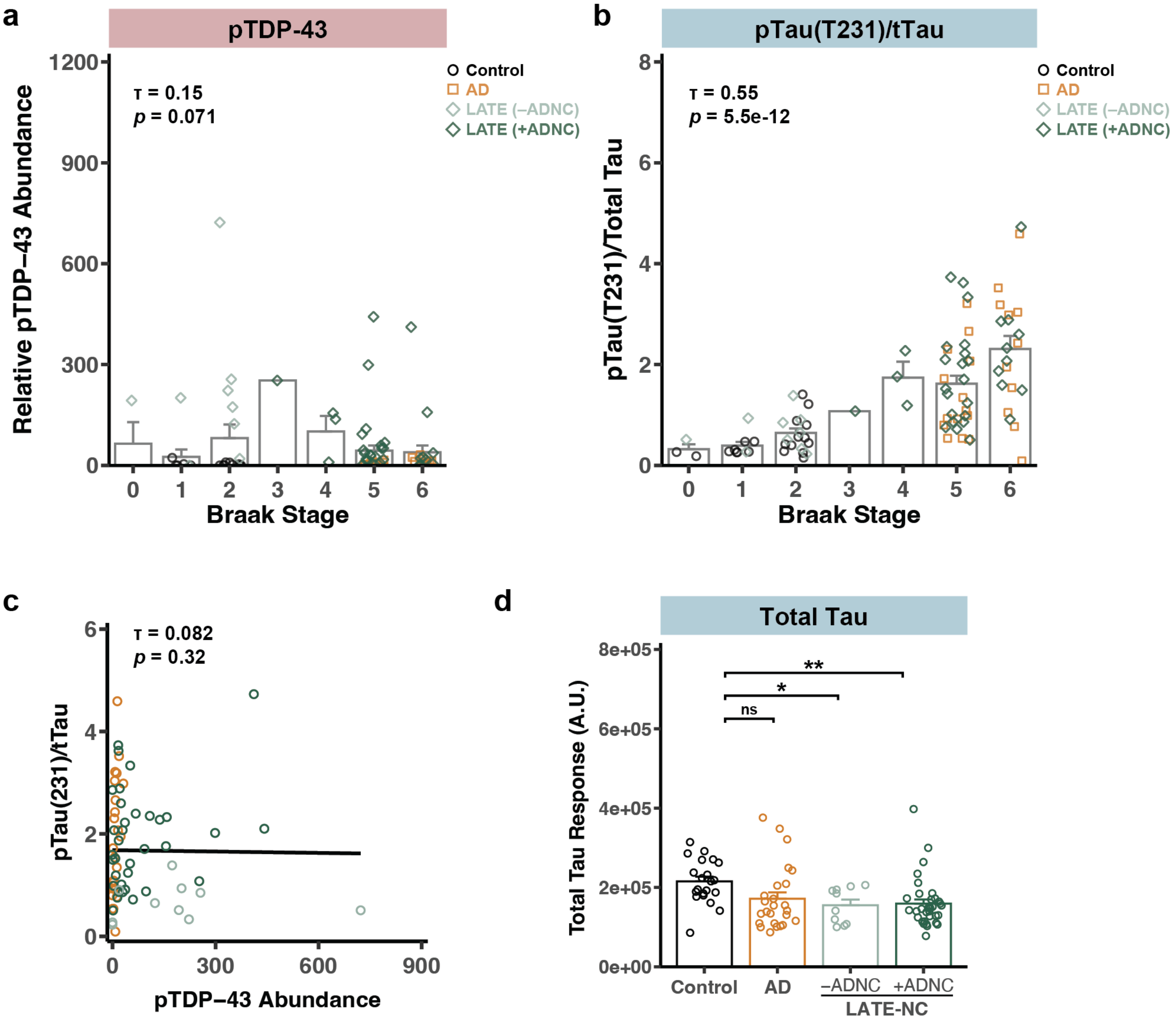

Supplementary Figure 3: Associations of pTau(231) and pTDP-43. a. Quantitative pTDP-43 abundance does not correlate with Braak Stage in our cohort. **b.** pTau(231)/tTau ratio correlates strongly with Braak Stage. **c.** pTDP-43 and pTau(231)/tTau do not correlate in AD and LATE-NC. **d.** Total tau is significantly reduced in LATE-NC with or without ADNC. (*a*-*c*) Kendall's τ Rank correlation. (*d*) One Way Analysis of Variance (ANOVA) followed by Dunnett's multiple comparison test

Supplementary Figure 4:

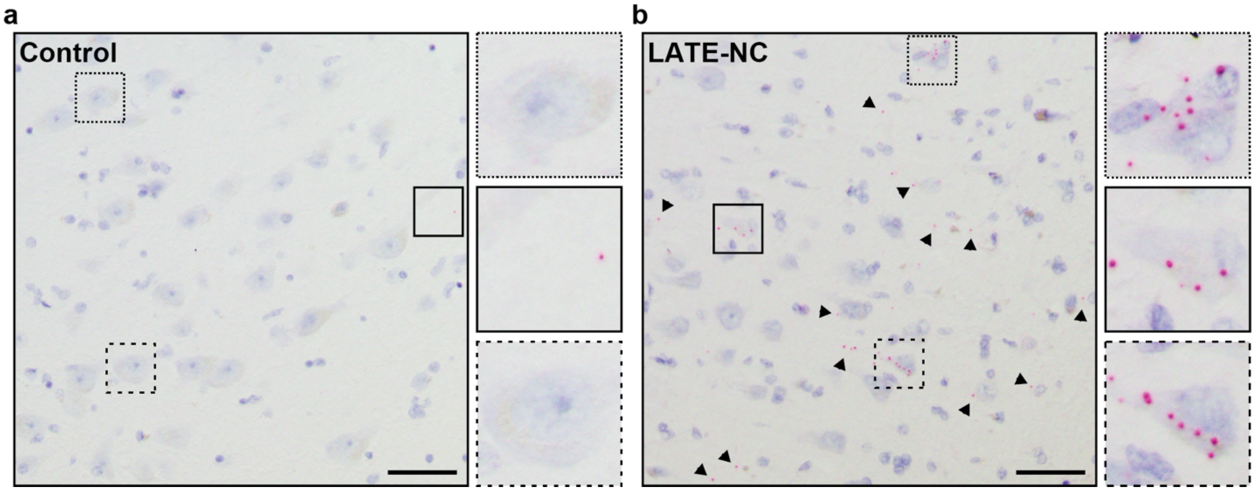

Supplementary Figure 4: Detection of t*STMN2* RNA molecules in LATE-NC by Basescope™ *in situ* hybridization assay. BaseScope™ *in situ* hybridization (ISH) assay in Control **(a)** and LATE-NC **(b)** patient hippocampal sections. Robust detection of intra- and extranuclear t*STMN2* RNA molecules were observed both in LATE-NC but not Controls. Very few red dots were observed in Control or AD tissue sections. Scale bar = 50μm.

SUPPLEMENTARY TABLES:

Supplementary Table 1: Detailed Neuropathological, Clinical, and Demographic Characteristics of Individual Cases

Sample #	Disease Group	Age at Onset	Age at Autopsy	Sex	Cause of Death	Brain Weight (g)	PMI (hrs)	Thal Phase	Braak Stage	CERAD Plaque	CERAD AD	ADNC Severity	Hipp. Sclerosis	Lewy Body	ApoE	Last Clin Dx	MMSE	pTDP-43 Abun.	Tau Ratio
1	AD	N/A	88	M	"End stage dementia"	1080	2		V		C = Definite AD	High likelihood	No	No				5.19	2.30
2	AD	81	86	F		1232	2.8	5	V	Frequent	C = Definite AD	High likelihood	No	No	e3/e4	DEMENTED	15	0.00	0.54
3	AD	73	82	M		1274	4.9	4	V	Frequent	C = Definite AD	High likelihood	No	No	e3/e4	DEMENTED	19	8.69	2.66
4	AD	77	84	M	GI Bled	1440	1.3	5	V	Moderate	C = Definite AD	High likelihood	No	No	e3/e3	DEMENTED	27	0.00	0.80
5	AD	90	92	M	CHF	1390	3.5	5	V	Moderate	C = Definite AD	High likelihood	No	No	e3/e3	MCI	23	0.00	0.99
6	AD	79	83	M	Multiple Medical - Vascular, diverticulitis, intestinal blockage, bowel penetration	1230	3.4	4	V	Frequent	C = Definite AD	High likelihood	No	Yes	e3/e4	DEMENTED	28	9.19	0.92
7	AD	63	73	F		940	4.4		VI		C = Definite AD	High likelihood	No	No	e3/e4	DEMENTED	4	1.19	1.05
8	AD	N/A	74	F	MI	1010	2.7		VI		C = Definite AD	High likelihood	No	No				8.19	0.09
9	AD	66	71	M	Dementia	1325	2.7	5	V	Moderate	B = CERAD probable	Intermediate likelihood	No	Yes	e3/e3	DEMENTED	13	2.69	0.54
10	AD	65	80	M	End stage AD	1020	1.8	5	V	Frequent	C = Definite AD	High likelihood	No	No	e4/e4	DEMENTED		16.19	2.07
11	AD	66	77	F	Natural	1029	4.2	5	VI	Frequent	C = Definite AD	High likelihood	No	Yes	e4/e4	DEMENTED		23.69	1.95
12	AD	72	80	F	Complications from fall resulting in broken neck; onventilator for 1.5 days	1260	3.7	5	V	Frequent	C = Definite AD	High likelihood	No	Yes	e3/e4	MCI	26	7.69	3.21
13	AD	65	74	F	AD	1020	1.9	5	VI	Frequent	C = Definite AD	High likelihood	No	No	e3/e4	DEMENTED	20	7.19	2.43
14	AD	66	90	F	End Stage AD	920	2.6	5	VI	Frequent	C = Definite AD	High likelihood	Yes	No	e2/e4	DEMENTED	0	19.19	3.52
15	AD	84	94	M	AD + Vascular disease	1030	3.8	5	VI	Frequent	C = Definite AD	High likelihood	No	No	e3/e3	DEMENTED	19	12.19	3.19
16	AD	73	76	F	Apirating, hard time catching breath	1150	2.3	3	VI	Frequent	C = Definite AD	High likelihood	No	Yes	e3/e4	DEMENTED	27	6.69	3.04
17	AD	79	83	F	Cancer and COPD	1280	4	5	V	Moderate	C = Definite AD	High likelihood	No	No	e4/e4	DEMENTED	26	0.00	0.52
18	AD	80	90	F	End Stage Dementia	1020	4.5	5	VI	Frequent	C = Definite AD	High likelihood	No	No	e3/e3	DEMENTED	2	31.69	2.98
19	AD	52	63	F	? Found deceased	1280	3.4	5	VI	Frequent	C = Definite AD	High likelihood	No	No	e3/e4	DEMENTED	96	12.69	0.77

20	AD	N/A	93	F	End Stage Heart Disease	1190	1.8	5	V	Moderate	B = CERAD probable	High likelihood	No	No	e3/e4	NORMAL	26	12.69	1.35
21	AD	60	62	F	End Stage dementia	1110	3.5	5	VI	Frequent	C = Definite AD	High likelihood	No	No	e4/e4	DEMENTED	21	6.19	1.54
22	AD	67	73	F	Cardiopulmonary Failure	1150	1.9	5	VI	Frequent	C = Definite AD	High likelihood	No	No	e3/e4	DEMENTED	17	14.19	4.59
23	AD	77	82	F	End Stage AD	1030	2.6	5	V	Frequent	C = Definite AD	High likelihood	No	Yes	e3/e4	DEMENTED	25	1.69	1.72
24	AD	86	93	F	Dementia; UTI	1070	4.8	5	V	Frequent	C = Definite AD	High likelihood	No	No	e3/e4	DEMENTED	27	3.19	1.09
25	AD	79	91	M	Acute Myloid Leukemia	1310	2.4	4	V	Moderate	B = CERAD probable	High likelihood	No	No	e3/e4	DEMENTED	28	2.19	0.93
26	Control	N/A	90	F	End stage Ovarian cancer with mets to abdomin	1045	2.2	2	II	None	No	Low likelihood	No	No	e2/e3	NORMAL	30	3.69	0.40
27	Control	N/A	88	M	CHF and Rena failure	1290	2.1		II		A = CERAD possible	Low likelihood	No	No	e3/e3	NORMAL	30	1.19	0.55
28	Control	N/A	94	M	s/p pneumonia? MI?	1455	15	5	I	Sparse	A = CERAD possible	Low likelihood	No	No	e3/e3	NORMAL	29	0.00	0.27
29	Control	N/A	80	F	Ovarian cancer with Mets	1200	4.4	1	I	None	No	No	No	No	e3/e3	NORMAL	28	0.00	0.26
30	Control	N/A	85	F		1043	2.1	0	II	None	No	No	No	No	e3/e3	NORMAL	30	2.19	0.44
31	Control	N/A	92	M	Lung Cancer	970	3.8	3	II	None	No	Low likelihood	No	No	e3/e3	NORMAL	30	0.19	0.28
32	Control	N/A	69	F	Stomach Cancer	980	2.2	3	I	None	No	Low likelihood	No	No	e3/e3	NORMAL	29	4.19	0.26
33	Control	N/A	68	F		1030	4	1	I	None	No	No	No	No	e3/e4	NORMAL	30	0.00	0.28
34	Control	N/A	71	M	End stage Liver Cancer	1495	2.6	3	0	None	No	Low likelihood	No	No	e3/e3	NORMAL	28	0.00	0.18
35	Control	N/A	90	F	Pneumonia	1240	3.7	4	II	None	No	Low likelihood	No	No	e3/e3	NORMAL	30	0.00	0.88
36	Control	N/A	86	M	Pneumonia, Upper GI, worn out - stress	1125	4	2	II	None	A = CERAD possible	Low likelihood	No	No	e3/e3	NORMAL	28	9.19	0.80
37	Control	N/A	91	M	Stroke	1450	4.3	5	II	None	No	Low likelihood	No	No	e3/e3	NORMAL	26	0.69	1.22
38	Control	N/A	81	M	Bladder Cancer with Lung Mets	1250	4.8	0	0	None	No	No	No	No	e3/e3	NORMAL	28	0.00	0.27
39	Control	N/A	83	F	Lung Cancer	1410	2.8	1	I	None	No	No	No	No	e3/e4	NORMAL	30	0.00	0.47
40	Control	N/A	85	F	Metastatic Uterine Sarcoma	1280	1.5	0	II	None	No	No	No	No	e2/e3	NORMAL	28	8.69	0.15
41	Control	N/A	83	F	COPD	1140	1.8	1	II	None	No	No	No	No	e3/e3	NORMAL	30	0.00	0.57
42	Control	N/A	85	F	Pulmonary Fibrosis	1320	3	3	II	None	No	Low likelihood	No	No	e3/e4	NORMAL	30	0.00	1.41
43	Control	N/A	79	F	Colon Cancer - PERF	1210	2	0	I	None	No	No	No	No	e3/e3	NORMAL	26	22.69	0.31
44	Control	N/A	86	F	Pancreatic Cancer	1320	1.8	3	I	None	No	No	No	No	e2/e3	NORMAL	30	0.00	0.47

45	Control	N/A	94	M	Widely metastatic cance (not brain)	1190	2.6		II		No	Low likelihood	No	No	e2/e3	NORMAL	27	0.00	0.42
46	Control	N/A	91	F		1100	2.7	2	II	None	No	Low likelihood	No	No	e2/e3	NORMAL	29	0.00	0.25
47	LATE (-ADNC)	88	94	F	CHF/Aspiration pneumonia/renal failure	1120	1.8		0		No	No	Yes	No	e2/e3	DEMENTED	19	193.19	0.51
48	LATE (-ADNC)	84	91	F	Failure to thrive	1080	2.9		II		A = CERAD possible	Low likelihood	Yes	No	e2/e3	DEMENTED	29	256.19	0.85
49	LATE (-ADNC)	89	100	M	Bladder Cancer	1112	1.9	0	II	Sparse	A = CERAD possible	Low likelihood	Yes	Yes	e3/e3	DEMENTED	16	173.69	1.38
50	LATE (-ADNC)	88	92	M	Cancer? Type	1429	2.8	1	II	None	No	Low likelihood	Yes	No	e3/e3	MCI	30	722.69	0.51
51	LATE (-ADNC)	N/A	91	M	"Bronchiectasis"/Breathing difficulty	1155	2.3	2	I	None	No	Low likelihood	Yes	No	e3/e3	NORMAL	28	0.00	0.27
52	LATE (-ADNC)	87	95	F		990	2	4	I	Sparse	A = CERAD possible	Low likelihood	Yes	No	e2/e3	DEMENTED	12	201.19	0.94
53	LATE (-ADNC)	N/A	101	F		1050	2.4	2	II	Sparse	A = CERAD possible	Low likelihood	Yes	No	e3/e3	NORMAL	27	123.69	0.65
54	LATE (-ADNC)	83	90	M	Pneumonia	1220	3.3	0	II	None	No	No	No	No	e2/e3	MCI	30	0.00	0.23
55	LATE (-ADNC)	86	90	M		1305	2.8	1	II	None	No	Low likelihood	Yes	No	e3/e3	DEMENTED	20	222.69	0.33
56	LATE (-ADNC)	71	79	F	Hypoxia due to lung failure	1230	1.9	1	II	None	No	Low likelihood	Yes	No	e2/e3	DEMENTED	20	19.69	0.90
57	LATE (+ADNC)	86	89	F	Failure to thrive	1150	3.2	4	V	Frequent	C = Definite AD	High likelihood	Yes	No	e2/e4	DEMENTED	24	108.69	2.35
58	LATE (+ADNC)	86	91	F	Pneumonia	1229	2.2	3	V	moderate	B = CERAD probable	Intermediate likelihood	Yes	No	e2/e3	MCI	28	26.19	1.01
59	LATE (+ADNC)	75	82	M	End stage AD, multiple medical issue including CCL	1140	3.6	4	V	Frequent	C = Definite AD	High likelihood	Yes	No	e3/e4	DEMENTED	1	17.69	3.62
60	LATE (+ADNC)	85	95	F	Renal Failure	1128	2.1	5	V	Moderate	C = Definite AD	High likelihood	Yes	No	e3/e3	DEMENTED	17	14.19	0.75
61	LATE (+ADNC)	63	74	M		1290	2.4	5	V	Frequent	C = Definite AD	High likelihood	Yes	No	e3/e4	DEMENTED	11	8.19	1.52
62	LATE (+ADNC)	80	89	M		1134	2.5	5	IV	Moderate	B = CERAD probable	Intermediate likelihood	Yes	No	e3/e4	DEMENTED	19	9.69	1.19
63	LATE (+ADNC)	90	98	F	CHF/Infection	991	2.3	5	V	Frequent	C = Definite AD	High likelihood	Yes	No	e3/e3	DEMENTED	17	51.69	3.33
64	LATE (+ADNC)	82	89	M	Heart Failure	1140	1.6	4	V	Frequent	C = Definite AD	Yes	Yes	No	e3/e4	MCI	27	59.69	0.72
65	LATE (+ADNC)	92	96	F	Breat Cancer and Dementia	1030	2.5	5	VI	Frequent	C = Definite AD	High likelihood	Yes	No	e2/e3	DEMENTED	24	21.19	2.89
66	LATE (+ADNC)	70	85	F		880	2.2	5	VI	Frequent	C = Definite AD	High likelihood	Yes	No	e2/e3	DEMENTED	10	0.00	2.86
67	LATE (+ADNC)	81	85	M	Prostate Cancer with bone mets & AD	1190	2.1	5	V	Frequent	C = Definite AD	High likelihood	Yes	No	e2/e4	DEMENTED	3	45.19	1.24
68	LATE (+ADNC)	89	95	M	Pneumonia	1290	3.3	1	V	Moderate,	B = CERAD probable	Intermediate likelihood	Yes	No	e3/e3	DEMENTED	26	29.69	0.86

69	LATE (+ADNC)	82	94	F	Advanced AD- Failure to thrive	940	3	3	V	Frequent	C = Definite AD	Intermediate likelihood	Yes	No	e3/e3	DEMENTED	96	442.19	2.10
70	LATE (+ADNC)	89	93	F	Failure to thrive	980	3.5	4	V	Frequent	C = Definite AD	High likelihood	Yes	No	e4/e4	DEMENTED	0	298.69	2.02
71	LATE (+ADNC)	N/A	91	F		1035	3.8	5	VI	Frequent	C = Definite AD	High likelihood	Yes	No				18.19	1.87
72	LATE (+ADNC)	85	92	F		890	2.8	4	VI	Frequent	C = Definite AD	High likelihood	Yes	No	e2/e3	DEMENTED	16	158.19	2.33
73	LATE (+ADNC)	74	90	F	Lung Cancer	920	2	1	IV	Sparse	A = CERAD possible	Low likelihood	Yes	No	e2/e3	DEMENTED	19	155.69	1.76
74	LATE (+ADNC)	70	81	M		1002	3	5	VI	Frequent	C = Definite AD	High likelihood	Yes	No	e4/e4	DEMENTED	4	0.00	1.59
75	LATE (+ADNC)	79	83	F	Cancer and AD	1030	3.2	4	VI	Frequent	C = Definite AD	High likelihood	No	No	e2/e4	DEMENTED	22	37.19	0.91
76	LATE (+ADNC)	63	73	F	End Stage aD	960	1.3	5	VI	Frequent	C = Definite AD	High likelihood	Yes	No	e4/e4	DEMENTED	2	411.69	4.73
77	LATE (+ADNC)	82	92	M	Cardiopulmonary Arrest	1360	5.2	4	III	None	B = CERAD probable	Intermediate likelihood	No	No	e3/e3	MCI	30	97.69	0.88
78	LATE (+ADNC)	71	82	F	Dementia	1080	3	5	VI	Frequent	C = Definite AD	High likelihood	Yes	No	e3/e3	DEMENTED	11	4.69	2.07
79	LATE (+ADNC)	62	83	F		1080	5.9	5	VI	Frequent	C = Definite AD	High likelihood	Yes	Yes	e3/e4	DEMENTED	0	1.19	1.49
80	LATE (+ADNC)	78	88	F		1020	3	5	V	Frequent	C = Definite AD	High likelihood	No	Yes	e3/e4	DEMENTED	14	93.19	1.71
81	LATE (+ADNC)	85	89	M	Stroke - On respirator for 5 days	1345	3	3	V	Sparse	A = CERAD possible	Intermediate likelihood	Yes	No	e3/e4	DEMENTED	18	51.19	1.42
82	LATE (+ADNC)	78	87	F	Stroke	1200	2.3	5	V	Frequent	C = Definite AD	High likelihood	No	Yes	e3/e4	DEMENTED	13	36.19	2.22
83	LATE (+ADNC)	85	94	F	End stage dementia	1040	1.9	4	V	Frequent	B = CERAD probable	High likelihood	No	No	e3/e4	DEMENTED	21	1.69	0.51
84	LATE (+ADNC)	93	98	F		1090	1.9	3	III	Sparse	A = CERAD possible	Intermediate likelihood	Yes	No	e3/e3	DEMENTED	21	252.69	1.08
85	LATE (+ADNC)	80	96	F		1060	1.6	3	IV	Moderate	B = CERAD probable	Intermediate likelihood	Yes	No	e3/e3	DEMENTED	1	137.69	2.28
86	LATE (+ADNC)	87	94	M	Dementia - Aspiration	1250	2.5	5	V	Moderate	C = Definite AD	High likelihood	Yes	No	e3/e3	MCI	24	3.19	0.99
87	LATE (+ADNC)	90	100	F		1220	2.9	5	V	Frequent	C = Definite AD	High likelihood	Yes	Yes	e3/e4	DEMENTED	21	67.69	2.40
88	LATE (+ADNC)	77	86	M	End Stage Dementia	1120	2.3	5	V	Moderate	B = CERAD probable	High likelihood	Yes	Yes	e3/e3	DEMENTED	0	18.69	0.86
89	LATE (+ADNC)	77	92	M	Infection/Sepsis	1100	2.1	5	VI	Frequent	C = Definite AD	High likelihood	Yes	No	e3/e3	DEMENTED	15	24.69	2.60
90	LATE (+ADNC)	74	83	F	Fall - Hematoma - 8 days in hospice - Death	990	2	5	V	Frequent	C = Definite AD	High likelihood	Yes	No	e3/e4	DEMENTED	10	16.69	3.73
91	LATE (+ADNC)	79	90	F	End Stage Dementia	1140	2.3	3	V	Moderate	B = CERAD probable	Intermediate likelihood	Yes	No	e3/e4	DEMENTED	26	29.69	2.07

AD = Alzheimer’s Disease; ADNC = AD Neuropathological Changes; LATE = Limbic-predominant Age-related TDP-43 Encephalopathy; Hipp. Sclerosis = Hippocampal Sclerosis; NIARI = National Institute on Aging – Reagan Institute; MMSE = Mini-Mental State Examination; pTDP-43 Abun. – Relative Abundance of pTDP-43 as measured by MSD immunoassay; Tau Ratio – pTau(231)/tTau Ratio as measured by MSD immunoassay

Supplementary Table 2: Custom Cryptic Exon TaqMan Probe Info

Cryptic Exon	Primer	Sequence	Assay ID
SYT7	Forward Primer	CGTGGGCGCAGTGAGAA	APGZPNN
	Reverse Primer	GGCAGGCGGCAGACT	
	Reporter Probe	AAGGCTATCAACTTCCC	
KALRN	Forward Primer	CGGAGTTTGTGCGACTTCCA	APKCDUH
	Reverse Primer	GGACCAAGGTTCACAAATAGCAAAA	
	Reporter Probe	AAGTGAGCAGCCATATTC	
ARGHAP32	Forward Primer	AAAGAGAGGGTGTTTGGTTGTGA	APMF7HA
	Reverse Primer	GTTCCAGTTTCAGGGAGATGGTT	
	Reporter Probe	TCCAGGCTTCAAAACC	
PFKP	Forward Primer	AGGCCGACTGGGAGAGT	APMF7EF
	Reverse Primer	GGGAGGCACCGATTTGGA	
	Reporter Probe	CCTGCAAGTGAAATAA	
UNC13A	Forward Primer	TGGCCCCGTACCATGTC	APRWN37
	Reverse Primer	ACCAGCATTTATTCAACAAACTAGTTCCT	
	Reporter Probe	CCAGGCAGCTCATGCA	
ELAVL3	Forward Primer	GCCTCAAATTACAGACGAAGACCAT	AP7DWP9
	Reverse Primer	GGTCACAGCCGGAGTCA	
	Reporter Probe	CAGTGTCACATGCACCTTG	
CAMK2B	Forward Primer	GGCTTCCACTACCTGGTCTTC	APCFA9U
	Reverse Primer	CAGCTGCTGACTGACTATTCCT	
	Reporter Probe	CAGTGTGCCCTGTTAGATC	
STMN2	Forward Primer	TGCACATCCCTACAATGGCTAAAA	APZTKFJ
	Reverse Primer	TCTACCTTTCTCTCGAAGGTCTTCTG	
	Reporter Probe	CCGAGTCCCATTGCTG	

Supplementary Table 3: Information of Meso-Scale Discovery (MSD) Immunoassays

	**Control**	**AD**	**LATE-NC** (-ADNC)	**LATE-NC** (+ADNC)
pTDP-43 (A.U.)	0 (0 – 22.7)	7.19 (0 – 31.7)	183.4(0 – 722.7)	29.69 (0 – 422.2)
pTau(231)/Total Tau	0.40 (0.15 – 1.41)	1.54 (0.09 – 4.59)	0.58 (0.22 – 1.73)	1.76 (0.50 – 4.73)

pTDP-43 = phosphorylated TDP-43; pTau = phosphorylated Tau; A.U. Arbitrary Units (MSD Response Units)
